# 

**Published:** 1897-05

**Authors:** 


Vol. LI. MAY, 1897. No. 5.

### THE DENTAL REGISTER

A MONTHLY JOURNAL.

Devoted to the Interests of the Profession.

EDITED BY

J. TAFT, D. D. S.

ASSOCIATE EDITORS:

WILL. H. WHITSLAR, D. D. S. N. S. HOFF, D. D. S.

PUBLISHED BY

SAM'L A. CROCKER & CO.,

OHIO DENTAL AND SURGICAL DEPOT,

35, 37 and 39 West Fifth Street, CINCINNATI, OHIO.

Entered at the Postoffice at Cincinnati, O., as second class mail matter.

TERMS: $2.00 PER ANNUM IN ADVANCE. SINGLE COPIES, 25 CTS.

